# Active Polylactide-poly(ethylene glycol) Films Loaded with Olive Leaf Extract for Food Packaging—Antibacterial Activity, Surface, Thermal and Mechanical Evaluation

**DOI:** 10.3390/polym17020205

**Published:** 2025-01-15

**Authors:** Sylwia Grabska-Zielińska, Ewa Olewnik-Kruszkowska, Magdalena Gierszewska, Mohamed Bouaziz, Marcin Wekwejt, Anna Pałubicka, Anna Żywicka, Beata Kaczmarek-Szczepańska

**Affiliations:** 1 Faculty of Chemical Technology and Engineering, Bydgoszcz University of Science and Technology, Seminaryjna 3, 85-326 Bydgoszcz, Poland; 2 Department of Physical Chemistry and Physicochemistry of Polymers, Faculty of Chemistry, Nicolaus Copernicus University in Toruń, Gagarina 7, 87-100 Toruń, Poland; olewnik@umk.pl (E.O.-K.); mgd@chem.umk.pl (M.G.); 3 Electrochemistry and Environmental Laboratory, National Engineering School of Sfax, University of Sfax, BP1173, Sfax 3038, Tunisia; mohamed.bouaziz@isbs.usf.tn; 4 Biomaterials Technology Department, Faculty of Mechanical Engineering and Ship Technology, Gdańsk University of Technology, 80-233 Gdańsk, Poland; marcin.wekwejt@pg.edu.pl; 5 Department of Laboratory Diagnostics and Microbiology with Blood Bank, Specialist Hospital in Kościerzyna, 83-400 Kościerzyna, Poland; apalubicka@op.pl; 6 Department of Microbiology and Biotechnology, Faculty of Biotechnology and Animal Husbandry, West Pomeranian University of Technology in Szczecin, Piastów 45, 70-311 Szczecin, Poland; anna.zywicka@zut.edu.pl; 7 Department of Cosmetic and Biomaterials Chemistry, Faculty of Chemistry, Nicolaus Copernicus University in Toruń, Gagarina 7, 87-100 Toruń, Poland; beata.kaczmarek@umk.pl

**Keywords:** olive leaf extract, polylactide, poly(ethylene glycol), active films, food packaging

## Abstract

As the demand for sustainable and innovative solutions in food packaging continues to grow, this study endeavors to introduce a comprehensive exploration of novel active materials. Specifically, we focus on characterizing polylactide-poly(ethylene glycol) (PLA/PEG) films filled with olive leaf extract (OLE; *Olea europaea*) obtained via solvent evaporation. Examined properties include surface structure, thermal degradation and mechanical attributes, as well as antibacterial activity. The results indicated a significant impact of the incorporation of OLE into this polymeric matrix, increasing hydrophobicity, decreasing surface free energy, and enhancing surface roughness, albeit with slight reductions in mechanical properties. Notably, these modified materials exhibited significant bacteriostatic, bactericidal and anti-adhesive activity against both *Staphylococcus aureus* and *Escherichia coli*. Consequently, PLA/PEG/OLE films demonstrated considerable potential for advanced food packaging, facilitating interactions between products and their environment. This capability ensures the preservation and extension of food shelf life, safeguards against microbial contamination, and maintains the overall quality, safety, and integrity of the packaged food. These findings suggest potential pathways for developing more sustainable and effective food packaging films.

## 1. Introduction

Active packaging is one of the innovative systems or technologies that are increasingly used nowadays in the food packaging industry. This allows interaction between products and their environment to maintain or prolong the shelf-life of food, protecting and ensuring their microbial purity while maintaining the quality, safety, and integrity of food [1,2]. Two types of active packaging can be distinguished: systems that absorb or emit substances [3,4]. In the first type of system, absorbers can eliminate unwanted smells, flavors, and undesirable substances such as ethylene or oxygen. Emitters can emit active substances with antioxidative or antimicrobial properties [5,6].

Polylactic acid (PLA) is a widely used polymer in packaging applications due to its renewable origins and environmentally friendly nature. Derived from lactic acid, which is obtained via the fermentation of renewable resources such as tapioca, sugarcane, or corn, PLA offers a sustainable alternative to synthetic polymers produced from non-renewable resources [7,8,9]. It is compostable and reduces landfill waste. Approved as Generally Recognized as Safe (GRAS) by the FDA (Food and Drug Administration), PLA is considered suitable for food packaging, meeting safety and sustainability requirements [7,8]. Additionally, PLA’s properties can be optimized through material modifications by adding other polymers or active substances, enhancing its physical performance for diverse applications [10,11,12,13]. For example, polyethylene glycol (PEG) is commonly used as a plasticizer to improve PLA’s flexibility and mechanical performance [14]. Additionally, incorporating plant extracts and substances with antibacterial properties enhances the functionality of PLA-based materials, making them more suitable for applications as active food packaging [10,11,12,13,15].

Currently, active packaging materials based on polymers with incorporated antimicrobial compounds are becoming popular among scientists and food technologists [16,17,18,19]. Essential oils [10,20,21], flavonoids [15,22,23,24], and plant extracts [16,17,18,25,26,27,28,29] are used as modifiers of active packaging materials. Tea tree (TTO), manuka, bergamot, lemongrass, rosemary, clove, myrtle, thyme, or cinnamon essential oils have been used to modify polylactide-based films [10,20,21,30]. The materials modified with TTO, obtained by a solvent evaporation method, were characterized by satisfactory antibacterial properties against *Staphylococcus aureus* and *Escherichia coli*. Additionally, the flexibility of materials was significantly improved by the incorporation of TTO and poly(ethylene glycol) into the PLA matrix [10]. Zhang et al. obtained polylactide films containing tea tree and manuka oil, and they proved enhanced properties of electrospun fibers in terms of both antibacterial and mechanical characteristics [21]. Bergamot, lemongrass, rosemary, and clove essential oils, as additives to polylactide films, improved the percentage elongation at break and decreased the glass transition temperature of the materials [20]. In turn, rosemary, thyme, and myrtle oils were responsible for improved crystallization and the thermal stability of modified films manufactured by solvent-casting [30]. Cinnamon and clove oil-modified PLA films exhibited a complete zone of inhibition against *Campylobacter jejuni* [31].

Flavonoids, including catechin, quercetin, and its derivatives, can be used as color indicators, natural stabilizers, and valuable antimicrobial and antioxidative additives [12,15,32,33]. The phenomenon of plant extracts as substances incorporated into polymeric matrices is related to their compatibility with the matrix, the origination of extracts, their antibacterial properties, their antioxidant characteristics, and the availability of plants as an excellent source of active substances [34].

It is commonly known that olive leaf extract exhibits promising antioxidant, antibacterial, antifungal, anti-inflammatory, antiviral, and anti-carcinogenic properties [35,36]. Different solvents and their mixtures can be used to obtain olive leaf extract: methanol, ethanol, water, acetone, dichloromethane, petroleum ether, chloroform–methanol, methanol–water, or ethanol–water [37,38,39,40,41,42,43].

Different films based on gelatin, carrageenan, chitosan, or PLA with the addition of olive leaf extract were made and characterized [41,44,45,46]. Usually, these materials exhibit antimicrobial and antioxidant properties with improved mechanical and thermal properties. In our previous study on similar materials, we analyzed the composition of olive leaf extract using LC-MS/MS and confirmed the presence of phenolic compounds, such as oleuropein, hydroxytyrosol, gallic acid, caffeic acid, rutin, and verbascoside [47], which is consistent with already published reports [48,49]. In the DPPH test, we demonstrated the antioxidant activity of OLE introduced into polylactide/PEG materials. Additionally, we analyzed the structural properties of the films using SEM and FTIR techniques. Furthermore, changes in the materials’ color and their water vapor permeation were evaluated [47]. The addition of OLE to PLA/PEG-based materials did not affect the WVPR (Water Vapor Permeation Rate); however, the color of the films strongly depended on the OLE content. Building of our previous finding [47], the current study includes additional analysis to evaluate the impact of OLE addition to PLA/PEG-based materials, along with microbiological testing. The novelty of the present study lies in the comprehensive evaluation of polylactide and poly(ethylene glycol) films enriched with olive leaf extract for potential use as food packaging materials. While the combination of PLA, PEG, and OLE has been studied previously [47], this work expands on this by incorporating additional, previously unexplored analyses. Specifically, this study presents the following: microbiological properties against *Staphylococcus aureus* and *Escherichia coli*—an assessment of the bacteriostatic, bactericidal, and anti-adhesive activity of these materials, which has not been extensively reported in earlier research; surface properties—novel measurements of the films’ contact angle, surface free energy, and roughness (atomic force microscopy), providing valuable insights into their functionality and interactions with potential food surfaces; and thermal properties—a deeper exploration of the thermal behavior of the films, critical for evaluating their suitability in various packaging applications.

These investigations contribute to a deeper understanding of the materials’ properties and their potential as active packaging solution. Further studies are necessary to explore the full potential of PLA/PEG materials modified with the addition of OLE in food packaging applications.

## 2. Materials and Methods

### 2.1. Materials

Polylactide (type 2002D, average molecular weight = 155,500 Da) in the form of pellets was delivered by Nature Works® (Minnetonka, MN, USA). Poly(ethylene glycol) (average molecular weight = 1500 Da) and chloroform were purchased from Sigma-Aldrich Company (Steinheim, Germany). Leaves from an olive tree (*Olea europaea* L., cv Chetoui) were sourced from Tunisia (Sfax-Taoues, semi-arid climate, collected in July 2019).

### 2.2. Olive Leaf Extract—Obtainment and Characterization

The olive leaves were harvested and dried for 3 min in a microwave dryer (1200 W), and the dried leaves were milled and stored in 4 °C before the analysis. Firstly, a basic analysis to characterize the extract was conducted, and the results are described in our previous report [47].

(1) The extract was prepared from 2.5 wt.% olive leaf powder dispersion in chloroform, and was stirred for 1 h at 30 °C;

(2) The resulting solution was filtered using filter paper (Filtres RS, 8–11 mm, Madrid, Spain), and the residual solvent was evaporated at 40 °C under a vacuum;

(3) The obtained extract was freeze-dried, and its composition was analyzed by LC-UV-MS/MS (Thermo Fisher Scientific, Waltham, MA, USA) according to the method described in detail elsewhere [50]. The results of LC-UV-MS/MS characterization are described in our previous report [47].

### 2.3. Polylactide Films Formation

The preparation method was very simple, combining extraction and film fabrication in a nearly one-step process, as described in our previous report [47]. The films were obtained by solvent evaporation method at room temperature and humidity. In short: an appropriate amount of olive leaf dust was poured with chloroform, mixed with a magnetic stirrer for three hours and filtered. Polylactide was weighed and added to chloroformic OLE, creating a 3 wt.% solution. After that, poly(ethylene glycol) was weighed and added to chloroformic PLA/OLE, making 5 wt.% solution and mixed with a magnetic stirrer for 3 h. The obtained solution was poured onto a glass Petri dish with a 14 cm diameter, and the samples were dried for 24 h. Films containing 1, 3 and 5 wt.% of extracts were prepared. Polylactide with poly(ethylene glycol) films were used as a control sample. (As shown in the Supplementary Materials Figure S1).

### 2.4. Contact Angle Measurements and Surface Free Energy Calculation

The contact angles of two liquids, diiodomethane (D) and glycerol (G), on the surface of obtained films were measured at room temperature and humidity. A DSA10 goniometer equipped with system drop-shape analysis (Krüss GmbH, Hamburg, Germany) was used in the study. Liquid drops were placed onto the polymer surface with a microsyringe. The dropped image was recorded by a video camera and subsequently digitized. The profile of a single drop was numerically solved and fitted by appropriate mathematical functions using instrument software. Each accepted result of contact angle is the average value of 5 measurements, whose precision was 0.2°. The surface free energy (SFE) and its polar and dispersive components were calculated using the Owens–Wendt method [51].

### 2.5. Atomic Force Microscopy (AFM)

Topographic images of films were obtained using a multimode scanning probe microscope with a NanoScope IIIa controller (Digital Instruments, Santa Barbara, CA, USA) operating in tapping mode, in air, and at room temperature. Surface images were acquired at a fixed resolution (512 × 512 data points) using a scan width of 5 μm with a scan rate of 1.97 Hz. Root mean square roughness (Rq), mean arithmetic deviation of the profile from the mean line (Ra), and the maximum distance between the highest and lowest points of the recorded images (Rmax) were calculated from 25 μm^2^ surface area using NanoScope Analysis software (Bruker Optoc GmbH, Ettlingen, Germany).

### 2.6. Thermogravimetric Analysis (TGA)

Thermogravimetric analysis (TGA) of the polylactide films with an addition of PEG and OLE was performed on Simultaneous TGA-DTA Thermal Analysis type SDT 2960 (TA Instruments manufacturer, London, UK). The measurements were conducted under an air flow from room temperature to 600 °C, at a heating rate of 10 °C/min, in a nitrogen atmosphere.

### 2.7. Mechanical Properties

The mechanical properties of the film were determined using the nanoindentation technique with NanoTest™ Vantage equipment (Micro Materials, Wrexham, UK). A three-sided diamond pyramidal indenter (Berkovich indenter; Micro Materials, Wrexham, UK) was used. The following parameters were set up: 58 μm indentation depth, 15 s unloading time and 5 s holding time under maximum force. Load–displacement curves were recorded for each sample (n = 10), and hardness (H) as well as reduced Young’s modulus (Er) were calculated using the Oliver and Pharr method using integrated software. Moreover, two mechanical parameters were calculated: the resistance of the material to elastic deformation, defined as H/Er, and the material’s ability to dissipate energy at plastic deformation, i.e., H^3^/Er^2^.

### 2.8. Antibacterial Properties

#### 2.8.1. Microorganisms and Cultivation Conditions

The antibacterial properties of modified and control specimens were determined using Gram-negative bacteria *Escherichia coli* (American Type Culture Collection, ATCC 8739 and ATCC 35218) and Gram-positive bacteria *Staphyloccocus aureus* (ATCC 6538 and ATCC 29213).

Before the experiment, *S. aureus* and *E. coli* were plated onto the BHIA (Brain Heart Infusion agar, BioMaxima, Lublin, Poland) and cultivated overnight at 37 °C. After the incubation, one colony-forming unit (CFU) of each microorganism was transferred to 10 mL of BHI and incubated overnight in the same culture conditions while shaking. Prior to testing, the films were soaked in 70% EtOH for one hour and then washed in a sterile phosphate-buffered solution (PBS, Sigma Aldrich, Steinheim, Germany).

#### 2.8.2. Bacteriostatic Activity

Bacteriostatic activity was evaluated by measuring the turbidity of cultured bacterial broth with the tested films (n = 5) according to the McFarland standards [52], assuming the existence of a direct relation between turbidity and the number of bacteria. Briefly, when the optical density of the bacterial suspension is 1.0 McFarland index (iMS), the number of bacteria is 3 × 10^8^ CFU mL^−1^. Bacteria with an initial concentration of 1.5 × 10^8^ CFU/mL were cultivated in 2 mL Trypticase Soy Borth (Merck, Poland) at 37 °C. Optical density was measured using DensiCHEK Plus (BioMerieux, Durham, NC, USA), and readings were taken every 0.5 h. The maximum measuring range of this device was 4 MSi.

#### 2.8.3. Antibacterial Activity

Antibacterial activity was evaluated according to ISO 22196:2011 [53], with slight modifications. Briefly, the tested films were transferred to a Petri dish. Next, 200 µL of bacterial suspension in concentration 3 × 10^5^ CFU/mL in BHI medium was applied at several points on 0.4 g of each tested sample and incubated for 24 h at 37 °C with high humidity. After incubation, the samples were transferred into 50 mL plastic tubes with 20 mL of PBS and vortexed for 30 s to remove the bacterial cells attached to the surface of the samples. The number of bacterial cells was determined by a quantitative plating method on BHI agar medium after 24 h of incubation at 37 °C.

#### 2.8.4. Anti-Adhesive Activity

Evaluation of bacterial adhesion to the film surfaces was performed by immersing and incubating the tested sample in a bacterial solution, followed by drying and fixing, covering the sample with a gold layer and evaluation with Scanning Electron Microscopy (SEM, FEI Company, Hillsboro, OR, USA). Bacteria strains with an initial concentration of 1 × 10^8^ CFU/mL were added to 30 mL of Tryptic Soy Bulion (Merck, Poznań, Poland) and incubated with films (n = 3) at 37 °C for 14 days.

### 2.9. Statistical Analysis

Statistical analysis of the data was completed using commercial software (GraphPad Prism 8.0.1.244, GraphPad Software, San Diego, CA, USA). The results were presented as mean ± standard deviation (SD) and were statistically analyzed using a one-way analysis of variance (one-way ANOVA). Multiple comparisons between the means were performed with the statistical significance set at *p* ≤ 0.05. Results from water content and antibacterial activity were subjected to statistical analysis.

## 3. Results and Discussion

### 3.1. Olive Leaf Extract—Obtainment and Characterization

Previously, phenolic compounds have been identified by LC-MS/MS using the negative ionization mode of Chemlali olive leaf cultivar extract, including LC- MS/MS parameters and MRM transitions [47]. The results showed that gallic acid, hydroxytyrosol, caffeic acid, β-hydroxyverbascoside III, quercetin 3-O-rutinoside (rutin), verbascoside, luteolin 7-O-glucoside, oleuropein hexoside I, isoverbascoside, apigenin 7-O-glucoside, oleuropein I, oleuropein II, 6′-O-[(2E)-2,6-dimethyl-8-hydroxy-2-octenoyloxy]-secologanoside, and jaspolyoside III have been found in the extract [47].

Additionally, films based on PLA/PEG with olive leaf extract were basically characterized in terms of their physicochemical properties [47]. The total content of phenolic compounds and the DPPH radical scavenging assay were conducted to characterize fabricated films.

Current research constitutes the continuation of previous research in terms of the surface, mechanical and microbiological characteristics of the obtained materials. We have already confirmed that the modification of films with OLE positively influences their antioxidant properties and their crystallinity. The full physicochemical, surface and antimicrobial characteristics are necessary to predict the potential of these materials as candidates for food packaging applications. Therefore, this article presents further research results to provide a comprehensive characterization of the properties of PLA/PEG/OLE films.

### 3.2. Contact Angle Measurements

Glycerol (G) and diiodomethane (D) contact angles were measured to estimate the effect of OLE on the hydrophilicity of the PLA/PEG film surface. Both values of the contact angle for G and D, as well as the SFE, and its dispersive and polar components of films’ SFE are listed in Table 1.

The SFE of materials used in food packaging is a crucial parameter influencing various interactions between the packaging material and its environment. It plays a significant role in determining the wettability, adhesion, and overall performance of the packaging material [54]. The SFE influences the interactions with liquids, such as water or oils, and impacts the adhesion of inks, coatings, or adhesives to the packaging surface. Additionally, the SFE can affect the tendency of food particles to adhere to or repel from the packaging material [54]. Our results showed that the addition of OLE had a significant effect on the film’s wettability and the values of contact angles. For both glycerol and diodomethane, significant differences were noticed in contact angle values when comparing materials modified with the addition of OLE (1%, 3%, 5%) with the unmodified, control PLA/PEG material. In the case of SFE (and its dispersive and polar components), the values decreased significantly after the addition of olive leaf extract (PLA/PEG/1OLE, PLA/PEG/3OLE, PLA/PEG/5OLE) in comparison to the control sample PLA/PEG. Apart from that, it can be observed that for the PLA/PEG films after the addition of 1, 3, or 5% of OLE, the changes in values of SFE, $\gamma_{S}^{d}$ and $\gamma_{S}^{p}$ were not as significant.

Generally, the differences between native films based on PLA/PEG and films modified with OLE addition resulted from the interactions between polymer (PLA), plasticizer (PEG), solvent (chloroform) and phenolic compounds, which are the components of the lipid fraction of olive leaves. Besides phenolic compounds, the lipid fraction of olive leaves consists of other bioactive ingredients (such as chlorophylls, tocopherols, β-carotene, squalene, triterpenes, sterols) and omega-3 fatty acids (especially α-linolenic fatty acids) [55]. These substances have a hydrophobic nature, and they can influence the surface properties of PLA/PEG/OLE films.

### 3.3. Atomic Force Microscopy (AFM)

**Figure 1:**
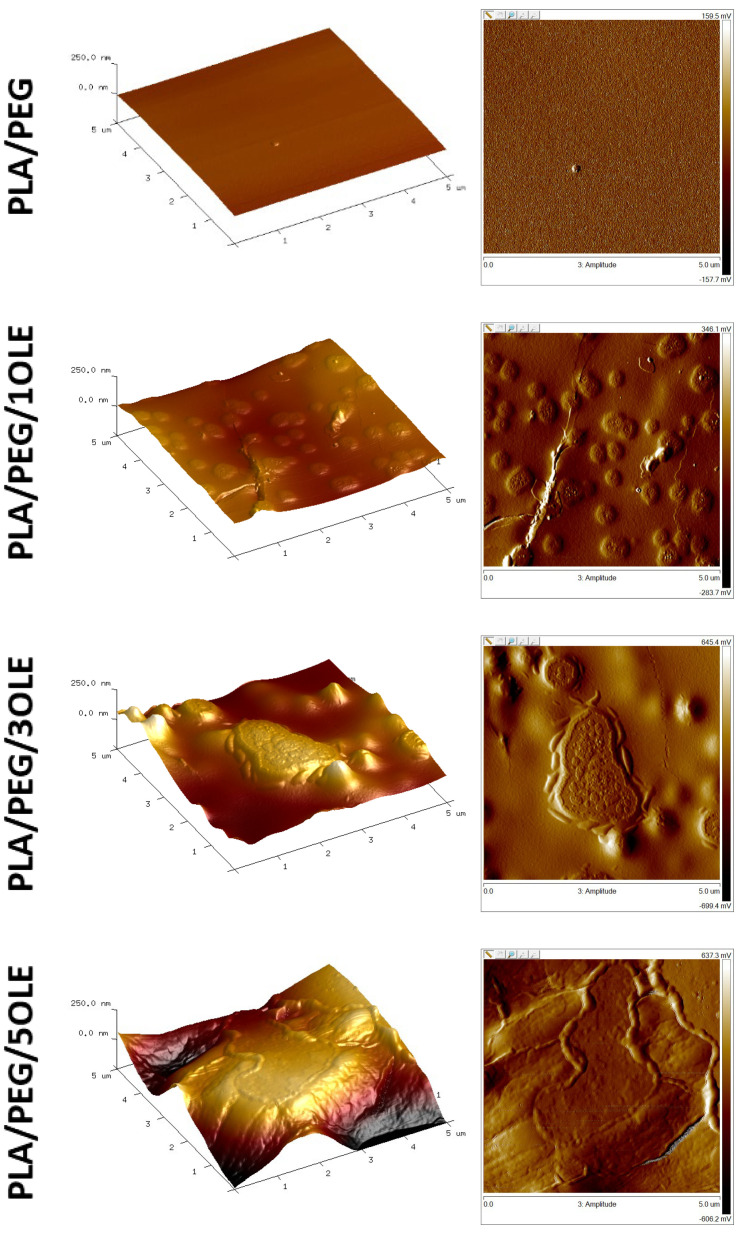
AFM topographic analysis of tested films.

Atomic Force Microscopy (AFM) was used to obtain the topographic images and the roughness parameters of prepared films (Figure 1 and Table 2). The neat PLA/PEG film showed a smooth and compact surface, while the films with loaded olive extract had significantly diverse topography. All calculated parameters, i.e., Rq, Ra and Rmax, increased with increasing OLE concentration. In both 2D and 3D images of the surface, it can be observed that as a result of modifications, hollows, bulges and surface irregularities were created on the surfaces. The tendency of changes in roughness parameters for materials modified with OLE is consistent with previous reports regarding PLA packaging films modified with extract of propolis [13], tea tree essential oil [10], quercetin [15] and birch tar [11].

On the one hand, the type of applied extract may have a significant impact on the topographies of the film [56], but on the other hand, the type of polymer blend itself may have an impact [57]. For example, Moraczewski et al. [58] prepared polylactide films with the addition of coffee, cocoa, and cinnamon extracts, and they noticed higher values of each roughness parameter when the extract addition was increased. Javadi et al. [59] reported that the AFM images of polylactide films with the addition of oregano essential oil (OEO) clearly demonstrate that the surface microstructure of the films is drastically affected by adding extract. These conclusions are similar to our results. When authors analyzed PLA films with OEO addition, they concluded that this trend could be associated with the more significant development of lipid aggregation and creaming during the drying step. In this experiment, oil was added to polylactide [59]. In our study, chloroform extract was used as a modifier for polylactide films. However, our samples of PLA/PEG/OLE film may contain, apart from phenolic compounds, lipid substances derived from olive leaves, which are soluble in chloroform.

### 3.4. Thermogravimetric Analysis (TGA)

**Figure 2:**
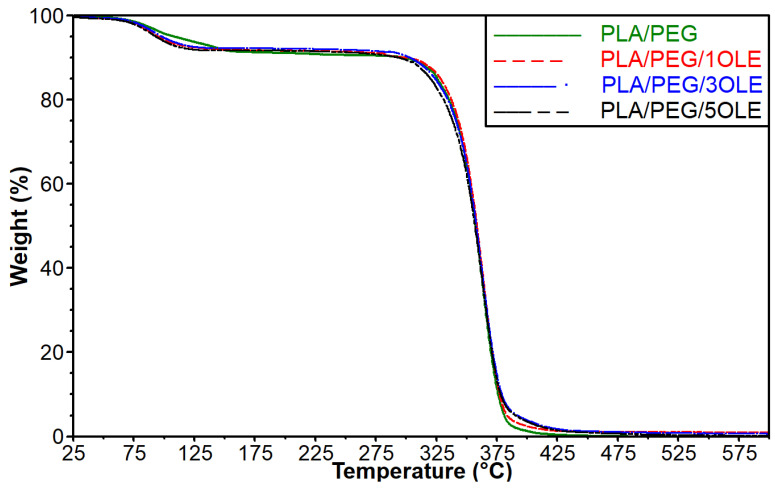
TG curves for polylactide-based films with an addition of PEG and OLE.

Thermogravimetric analysis was performed to establish the influence of the additive on the thermal stability of the obtained materials. The recorded data are shown in Figure 2, and the values of temperature at 5, 10, and 50% of mass loss of the analyzed materials are depicted in Table 3. It was observed that the thermal decomposition of all tested samples started below 100 °C. This phenomenon can be related to the evaporation of the solvent, i.e., chloroform, which was also observed in the case of our previous films based on PLA [10]. Moreover, additives in the form of olive leaf extract contain some volatile components that can evaporate at increased temperature [42,60]. The composition of the applied extract was described in our previous work [47]. It should be stressed that the lowest value of temperature at 5% mass loss was observed for film with an addition of 5% OLE. It is also worth noticing that the difference between temperature for PLA/PEG material and sample filled with 5% of extract exceeds 15 °C. After the evaporation of chloroform and volatile components present in the extract, the differences between temperatures at 10% of mass loss for individual materials are significantly reduced. As a result, it can be observed that the ΔT between the control sample and the material with the addition of 5% OLE is equal to 4 °C. Moreover, it should be noted that in the case of films containing smaller amounts of OLE (1 and 3%), the T_10%_ reached higher values than the PLA/PEG film. The other fact that must be considered is the difference between T_10%_ and T_5%_ values for particular samples. It is interesting that for a film consisting of PLA and PEG, the value of T_10%_ is approximately 189 °C higher than the T_5%_ value, while for materials with added OLE extract, the differences between the T_10%_ and T_5%_ values are significantly higher and are in the range between 200 and 204 °C. The obtained results suggest that the introduced extract, after the initial evaporation of volatile components, also contains components that are more thermally stable than the components of the PLA/PEG system. Further analysis of TG and T_50%_ values did not show significant differences in the behavior of the tested materials. In summary, it can be assumed that the addition of OLE, after the evaporation of the volatile compounds, can inhibit the decomposition of the formed materials.

### 3.5. Mechanical Properties

The mechanical properties of obtained films were evaluated using nanoindentation techniques, and the results are shown in Table 4. In food packaging applications, the mechanical properties of a tested material are essential for the maintenance of its structural integrity and enable functionality [61]. Both hardness and Young’s Modulus decreased with an increase in the content of olive leaf extract. By observing the microstructures of the modified films using SEM (available in [47]), significant changes in structure that may cause a reduction in mechanical properties were found (Table 4). These changes are associated with internal discontinuities, pores, and cavities, as well as the occurrence of phase separation between components, which was also observed in AFM results. However, only the additive with a concentration equal to 5% had a statistically significant effect on mechanical properties. Moreover, the impact of the modification on two indentation parameters was analyzed–resistance to elastic deformation (H/Er) and the ability to dissipate energy at plastic deformation (H^3^/Er^2^). Nevertheless, no statistically significant differences for these parameters were found. Hence, it can be concluded that the addition of up to 3% OLE has no significant effect on the mechanical properties of the obtained films.

The weakening of the film’s Young’s Modulus after different extract incorporation was previously observed in the literature. For example, López de Dicastillo et al. applied *murta* fruit extract (1% *w*/*v*) into methylcellulose/PEG films and found that they had a plasticizing effect [62]. Similarly, Kumar et al. observed that the mechanical properties of films decreased after incorporating pineapple peel extract into PVA–corn starch material [63]. This is consistent with the observations from our study. The increase in plasticity and, consequently, the decrease in Young’s modulus and hardness could be the results of the fact that in the olive leaf extract, in addition to phenolic compounds with antioxidant properties, there are also other compounds (as described in Section 3.1. *Contact Angle Measurements*, regarding contact angle and surface free energy of materials), which have a hydrophobic character. However, Ayana et al. evaluated the addition of OLE (up to 3%) into methylcellulose and concluded that tensile strength was improved due to additional cross-linking by aglycone [64]. This is the opposite trend to our study, but this is because no cross-linking agents were used in the preparation of our materials.

### 3.6. Bacteriostatic, Bactericidal and Antiadhesive Activity

The bacteriostatic activity of modified PLA/PEG films was assessed, and the obtained results are summarized in Table 5 and Table 6. The addition of OLE showed effective bacteriostatic effects against *Staphylococcus aureus* and *Escherichia coli* bacteria. Along with the increase in the content of OLE in films, a trend related to the slowdown in bacterial multiplication was observed.

Analyzing the results after 4.5 h of incubation for *S. aureus,* it can be stated that the additive inhibited bacterial growth by approximately 11%, 25% and 30% for 1%, 3% and 5% OLE concentrations, respectively. This corresponds to a reduction in the number of bacteria in solution (e.g., for 5OLE about 3.21 × 10^8^ CFU mL^−1^). Similarly, for the *E. coli*, analyzing the results after 2 h, the additive inhibited bacterial growth by approximately 0.4%, 18% and 22% for 1%, 3% and 5% OLE concentrations, respectively. For 5OLE, the reduction in the number of bacteria was about 1.8 × 10^8^ CFU mL^−1^. It can be concluded that the applied modification is more effective against *S. aureus* (Gram-positive bacteria) than *E. coli* (Gram-negative bacteria), and the most effective film regarding antibacterial infection is PLA/PEG/5OLE.

#### 3.6.1. Antibacterial Activity

**Figure 3:**
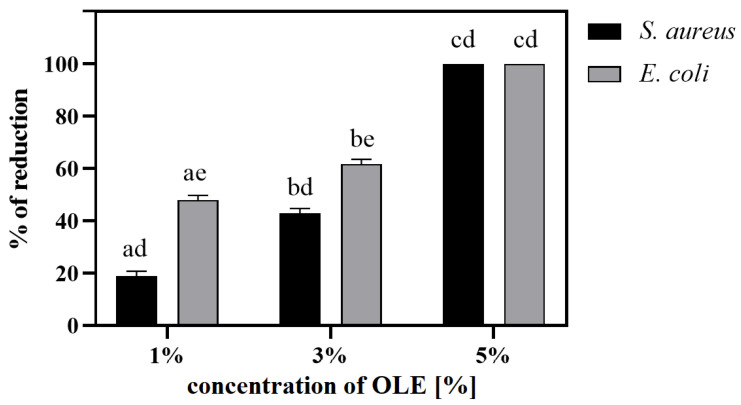
The antibacterial properties of PLA/PEG/OLE after 24 h of incubation. Data are presented as the mean of % of reduction compared to control ± standard error of the mean; values with different letters are significantly different (*p* < 0.05): a, b, c—statistically significant differences between the sample; d, e—statistically significant differences between the strains.

An additional study was carried out to check the potential antibacterial activity of the modified films (Figure 3), which showed that the OLE additive has bactericidal properties. At lower concentrations (up to 3% OLE), a higher reduction was found for *E. coli*, while in the case of 5% of the OLE additive, a 100% bactericidal effect was demonstrated for both bacteria.

**Figure 4:**
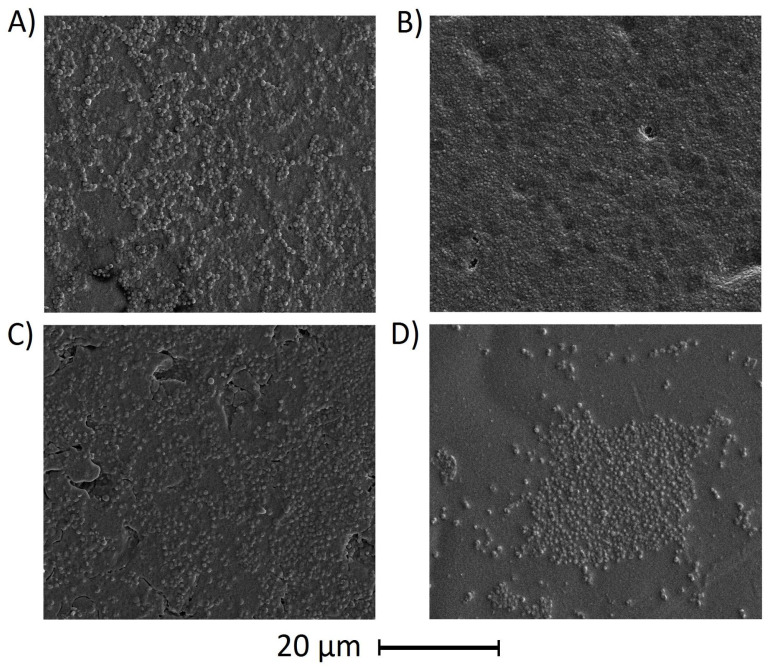
The comparison of bacterial adhesion to the film surface after 14 days of incubation in a bacterial suspension. (**A**) PLA/PEG–*S. aureus*; (**B**) PLA/PEG/5OLE–*S. aureus*; (**C**) PLA/PEG–*E. coli*; (**D**) PLA/PEG/5OLE–*E. coli* (the pictures are representative of three experiments).

PLA/PEG-based materials do not show any bioactive properties [65]; hence, various additives should be introduced to improve these properties [66,67]. Here, olive leaf extracts were added to films, causing an increase in the contents of various polyphenols [48], such as gallic acid, caffeic acid, hydroxytyrosol, β-hydroxyverbascoside III, quercetin 3-O-rutinoside, verbascoside, luteolin 7-O-glucoside, oleuropein hexoside I, isoverbascoside, apigenin 7-O-glucoside, 6′-O-[(2E)-2,6-dimethyl-8-hydroxy-2-octenoyloxy]-secologanoside, jaspolyoside III and oleuropein I and II. It has been proven in numerous studies that these compounds have bioactive properties, including antibacterial activity [49]. In our previous study [47], we confirmed the presence of the above-mentioned phenolic compounds and their various total contents in the developed films: 0.349, 0.498 and 0.637 mg/mL for PLA/PEG/1OLE, 3OLE and 5OLE, respectively. Now, it has been confirmed that this modification contributes to obtaining the antibacterial activity of PLA/PEG materials, especially effective with a 5% OLE additive and short incubation time. Prepared films exhibit surface bactericidal properties against *S. aureus* and *E. coli* (up to 24 h; Figure 3) and simultaneously release active substances into the liquid environment already in the first hours of incubation (0.5–4/6 h; Table 5 and Table 6). However, in the more extended period of incubation in a bacterial environment (14 days), a bacterial biofilm of both bacteria was observed on their surfaces (Figure 4). The films showed greater surface antibacterial activity against *E. Coli*, while in the case of testing in liquid solution, against *S. aureus*.

Bastante et al. confirmed that both *S. aureus* and *E. coli* presented similar sensitivity to OLE [68]. However, Sudjana et al. suggested that one or more compounds in olive leaf extract may have a specific effect against bacteria cell walls (Gram +); hence, the antibacterial activity may vary [69]. Also, Musella et al. modified chitosan film by OLE (up to 30%). However, they did not improve the antibacterial properties, and chitosan itself was the most effective [46]. Hence, due to the differences in the results, attention should also be paid to the differences in the procedure of extract preparation, which significantly affects their properties [70], such as, for example, the solvent used or parameters of extraction. However, apart from the difference between the bacterium types, there are examples in the literature confirming the effectiveness of OLE as a modification for films for food packaging applications, similar to our conclusion. Similarly, Ayana et al. applied OLE modification (up to 3%) into methylcellulose and confirmed its effectiveness against *S. aureus* [64]. Also, Albertos et al. found that the addition of 5.63% OLE to gelatin films is an optimal and effective antibacterial modification against *L. monocytogenes* and enables their potential use in cold-smoked fish preservation [44].

#### 3.6.2. Antiadhesive Properties

The PLA/PEG and PLA/PEG/5OLE films were incubated in bacterial suspension, *S. aureus* or *E. coli*, for 14 days to assess their anti-adhesive properties. The results of surface SEM observations after the experiment are shown in Figure 4. The presence of bacteria and their bacterial biofilm was observed on all the examined films. The presence of bacteria was observed on all surfaces, and in the case of *S. aureus,* a clearly visible bacterial biofilm was found. Hence, it can be assumed that OLE addition (up to 5%) does not affect the biofilm formation of bacteria in PLA/PEG films.

## 4. Conclusions

Active antimicrobial materials are essential in ensuring the desired quality of food packaging, meeting the expectations of the contemporary food industry. Considering our previous outcomes for the physicochemical characterization of PLA/PEG/OLE films, as well as the research presented here, it may be inferred that the proposed modification represents an exceptionally intriguing direction in the development of active packaging. The primary advantage lies in its effective antibacterial ability, enabling the protection of food from bacterial influences, which is in line with the growing trend of incorporating active components. The addition of OLE (in concentrations up to 5%), in comparison to control PLA/PEG films, significantly influences the surface energy—decreasing by ~7.7–9.1 mJ/m^2^ and leading to a more diversified structure—as indicated by an increase in Rmax by ~2–4 times. Importantly, this modification does not impact thermal degradation. From the perspective of potential application, the most optimal modification appears to be the 3% OLE addition, which provides active properties without adversely affecting the mechanical characteristic. In summary, the incorporation of olive leaf extract represents an exceedingly promising approach in the context of active packaging. The proposed PLA/PEG/OLE films have the potential to significantly improve the shelf life and quality of packaged food, marking a substantial contribution to the food packaging industry.

## Figures and Tables

**Table 1:** Values of contact angles, surface free energy (SFE) and its dispersive $\gamma_{S}^{d}$ and polar $\gamma_{S}^{p}$ components (n = 5; * significantly different from control sample—PLA/PEG (*p* < 0.05)).

Sample	Contact Angle [°]		SFE [mJ/m^2^]	$\gamma_{S}^{d}$ [mJ/m^2^]	$\gamma_{S}^{p}$ [mJ/m^2^]
	G	D			
**PLA/PEG**	74.62 ± 0.62	48.55 ± 0.23	35.31 ± 0.09	30.39 ± 0.05	35.31 ± 0.09
**PLA/PEG/1OLE**	85.72 ± 0.91 *	62.20 ± 1.65 *	27.17 ± 0.45 *	24.24 ± 0.35 *	27.17 ± 0.45 *
**PLA/PEG/3OLE**	99.27 ± 1.06 *	64.34 ± 0.88 *	26.25 ± 0.21 *	26.06 ± 0.20 *	26.25 ± 0.21 *
**PLA/PEG/5OLE**	96.23 ± 0.70 *	61.54 ± 2.27 *	27.63 ± 0.55 *	27.26 ± 0.50 *	27.63 ± 0.55 *

**Table 2:** Roughness parameters (Ra, Rq, Rmax) of PLA/PEG and PLA/PEG/OLE films; the differences between the results of all samples were statistically significant.

Specimen	Ra [nm]	Rq [nm]	Rmax [nm]
**PLA/PEG**	4.79 ± 0.08	5.87 ± 0.18	35.47 ± 0.46
**PLA/PEG/1OLE**	21.90 ± 1.82	25.40 ± 1.29	138.93 ± 3.90
**PLA/PEG/3OLE**	45.10 ± 1.56	54.60 ± 3.09	280.67 ± 8.02
**PLA/PEG/5OLE**	77.20 ± 3.22	94.60 ± 4.74	461.67 ± 7.02

**Table 3:** Values of temperatures at different mass loss and the difference between temperatures at 5% and 10% of mass loss.

Sample	Temperature (°C) at Mass Loss			ΔT (T_10%_-T_5%_)
	T_5%_	T_10%_	T_50%_	
**PLA/PEG**	108.82	297.75	357.43	188.93
**PLA/PEG/1OLE**	95.52	299.74	358.55	204.22
**PLA/PEG/3OLE**	98.56	303.05	358.07	204.49
**PLA/PEG/5OLE**	93.53	293.47	356.84	199.94

**Table 4:** The mechanical properties of the tested films (n = 10; * significantly different from control sample—PLA/PEG (*p* < 0.05)).

Sample	Hardness (H; MPa)	Reduced Young’s Modulus (Er; MPa)	H/Er	H^3^/Er^2^ (MPa)
**PLA/PEG**	21.3 ± 1.5	218.9 ± 17.0	0.097 ± 0.003	0.201 ± 0.030
**PLA/PEG/1OLE**	20.2 ± 0.8	204.6 ± 10.3	0.091 ± 0.005	0.196 ± 0.015
**PLA/PEG/3OLE**	19.8 ± 0.6	208.2 ± 14.7	0.095 ± 0.016	0.179 ± 0.028
**PLA/PEG/5OLE**	18.3 ± 1.2 *	172.1 ± 7.4 *	0.106 ± 0.011	0.207 ± 0.017

**Table 5:** The *Staphylococcus aureus* growth inhibition determined by McFarland standard values (MSi) during incubation with the tested films (n = 3; * significantly different from control sample—PLA/PEG (*p* < 0.05)).

McFarland Standard Values Specifying the Number of *Staphylococcus aureus* Bacteria				
Time [h]	PLA/PEG	PLA/PEG/1OLE	PLA/PEG/3OLE	PLA/PEG/5OLE
**0**	0.3			
**0.5**	0.53 + 0.02	0.53 + 0.02	0.52 + 0.01	0.50 + 0.02
**1.0**	0.86 + 0.01	0.84 + 0.01	0.75 + 0.01 *	0.73 + 0.01 *
**2.0**	1.74 + 0.01	1.42 + 0.02 *	1.12 + 0.02 *	1.07 + 0.04 *
**3.0**	2.10 + 0.01	1.72 + 0.02 *	1.53 + 0.02 *	1.46 + 0.01 *
**4.0**	3.22 + 0.02	2.65 + 0.02 *	2.38 + 0.04 *	2.29 + 0.03 *
**4.5**	3.49 + 0.02	3.09 + 0.02 *	2.61 + 0.02 *	2.42 + 0.02 *
**5.0**	>4	3.66 + 0.02 *	3.35 + 0.03 *	3.11 + 0.02 *
**5.5**		>4	3.72 + 0.05 *	3.49 + 0.03 *
**6.0**			>4	>4

**Table 6:** The *Escherichia coli* growth inhibition determined by McFarland standard values (MSi) during incubation with the tested films (n = 3; * significantly different from control sample—PLA/PEG (*p* < 0.05)).

McFarland Standard Values Specifying the Number of *Escherichia coli* Bacteria				
Time [h]	PLA/PEG	PLA/PEG/1OLE	PLA/PEG/3OLE	PLA/PEG/5OLE
**0**	0.3			
**0.5**	0.57 + 0.02	0.57 + 0.02	0.52 + 0.01 *	0.52 + 0.01 *
**1.0**	1.42 + 0.02	1.41 + 0.01	1.16 + 0.02 *	1.12 + 0.01 *
**2.0**	2.61 + 0.01	2.60 + 0.02	2.14 + 0.04 *	2.01 + 0.04 *
**3.0**	>4	>4	3.36 + 0.02 *	3.34 + 0.01 *
**4.0**			>4	>4

## Data Availability

The data presented in this study are available in the manuscript or are on request from the corresponding author.

## Supplementary Materials

The following supporting information can be downloaded at: https://www.mdpi.com/article/10.3390/polym17020205/s1, Figure S1: Real photo of polylactide/poly(ethylene glycol)/olive leaf extract solutions to obtain thin packaging films.
