# Supplementary material for: Active Polylactide-poly(ethylene glycol) Films Loaded with Olive Leaf Extract for Food Packaging—Antibacterial Activity, Surface, Thermal and Mechanical Evaluation
Source: Polymers (Basel). 2025 Jan 15;17(2):205. doi: 10.3390/polym17020205 (PMC11769166; doi:10.3390/polym17020205)
Supplement: Supplementary file 1 [file polymers-17-00205-s001.zip › polymers-3396527-supplementary.pdf]

polymers

Supplementary Materials

Active Polylactide-Poly(Ethylene Glycol) Films Loaded with Olive Leaf Extract for Food Packaging – Antibacterial Activity, Surface, Thermal and Mechanical Evaluation

Sylwia Grabska-Zielińska, Ewa Olewnik-Kruszkowska, Magdalena Gierszewska, Mohamed Bouaziz, Marcin Wekwejt, Anna Pałubicka, Anna Żywicka and Beata Kaczmarek-Szczepańska

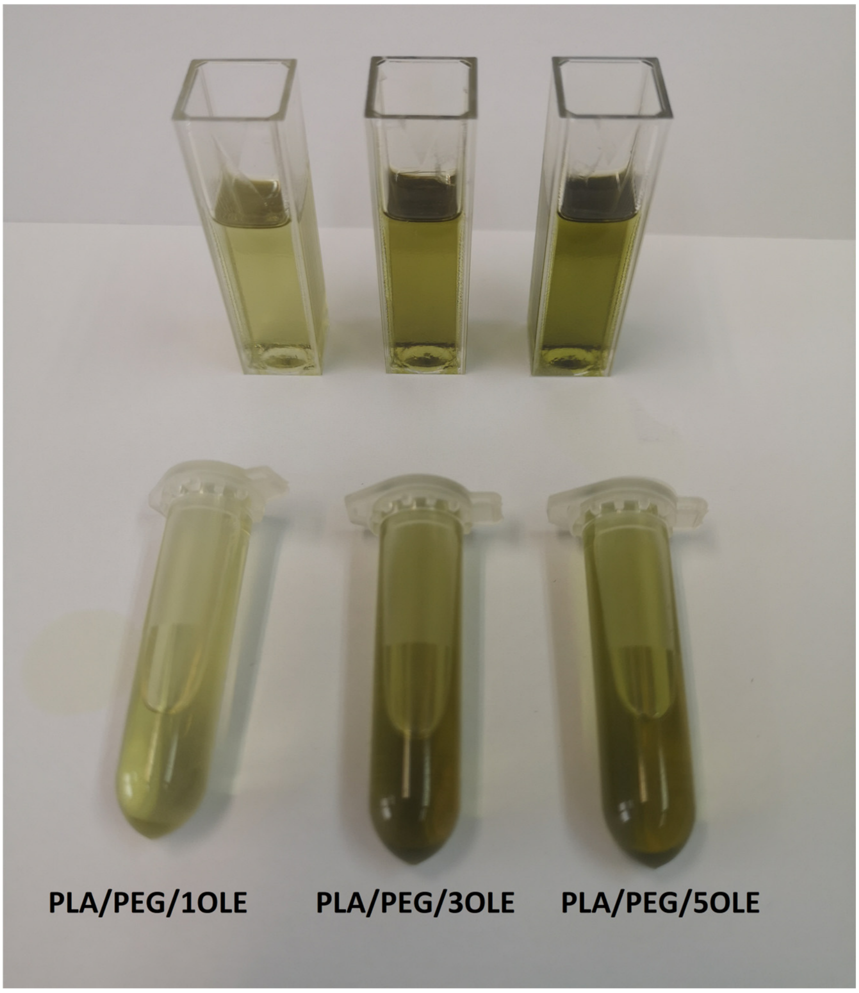

Figure S1. Real photo of polylactide/poly(ethylene glycol)/olive leaf extract solutions to obtain thin packaging films.
